# The Fellowship of Privileged Scaffolds—One Structure to Inhibit Them All

**DOI:** 10.3390/ph14111164

**Published:** 2021-11-16

**Authors:** Marcin Skoreński, Marcin Sieńczyk

**Affiliations:** Department of Organic and Medicinal Chemistry, Faculty of Chemistry, Wroclaw University of Science and Technology, Wybrzeze Wyspianskiego 27, 50-370 Wrocław, Poland; marcin.skorenski@pwr.edu.pl

**Keywords:** privileged structures, enzyme inhibitors, antivirals

## Abstract

Over the past few years, the application of privileged structure has emerged as a powerful approach to the discovery of new biologically active molecules. Privileged structures are molecular scaffolds with binding properties to the range of different biological targets. Moreover, privileged structures typically exhibit good drug-like properties, thus assuring more drug-like properties of modified compound. Our main objective is to discuss the privileged structures used for the development of antiviral agents.

## 1. Introduction

**Figure 1:**
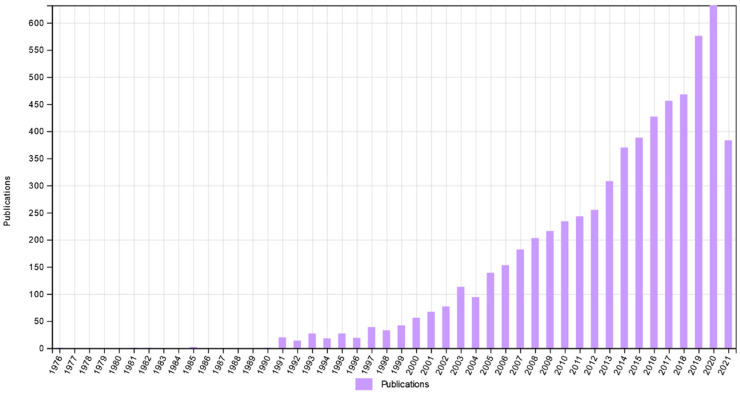
Numbers of publications over time when searching “privileged structure” as topic term (according to Web of Science, date: 27 September 2021).

The term ‘privileged structure’ was first coined in 1988 by Evans et al., who used it to describe repeating structural motifs as useful templates for discovering new biological active molecules [1]. During their early studies, they had noticed that particular chemical structures are able to bind with equal affinity to more than one type of receptor. Subsequently, the approach where sensible modifications of privileged structures could lead to the proposal of new potent agonists or antagonists of the receptors. Since then, the number of studies in the field of medicinal chemistry that employed this methodology has been systematically growing, reaching now 6285 records according to Web of Science (Figure 1).

In the context of the ‘privileged structure’ term, it is worth addressing the issue of pan-assay interference compounds (PAINS). These molecules bind to the target but their activity does not depend on a specific, drug-like interaction between molecule and protein. Thus, there is a risk of mistake identification of chemical scaffold as a privileged structure while it is PAINS [2,3,4,5,6]. Currently, 400 structural classes of PAINS have been identified, but the most frequent PAINS encompasses 16 categories [7,8]. In some cases, PAINS are able to react with protein in the specific, drug-like mode and could serve as a starting template for structure optimisation. To exclude incorrect hit identification, researchers should check the literature carefully and look into the similarities between reported PAINS. It would be beneficial to conduct at least two types of activity assays to identify hits [8].

Despite possible pitfalls associated with PAINS, an increasing number of new inhibitors targeting diverse molecular targets and the availability of improved data analysis tools prompt a conclusion that the privileged structure-based approach will be applied in a larger number of studies. Here, we discuss privileged structures which were identified as highly useful in the development of novel antiviral agents acting at various molecular levels. We also describe new scaffolds which have not been identified in scientific literature so far. We have only dealt with scientific reports published after 2009.

## 2. Diaryl Ether (DE)

The diaryl ether (DE) motif is present in many drugs which have already been approved by the FDA (Food and Drug Administration). Roxadustad, Ibrutinib and Sorafenib are only three representatives thereof [9,10,11]. This structural scaffold bearing two aromatic rings and flexible oxygen bridge displays high hydrophobicity, which is why DE improves cell membrane penetration of the final molecule and its solubility in lipids. An incorporation of DE into the target biologically active compound ensures good metabolic stability of the new molecule [12]. Recently, Tao Chen et al. and Kini et al. published excellent, comprehensive reviews about the diaryl ether scaffold present in various drugs and agrochemicals [13,14]. Here, we would like to focus on antiviral agents containing DE moiety in their structure.

### 2.1. Anti-HIV Agents

**Figure 2:**
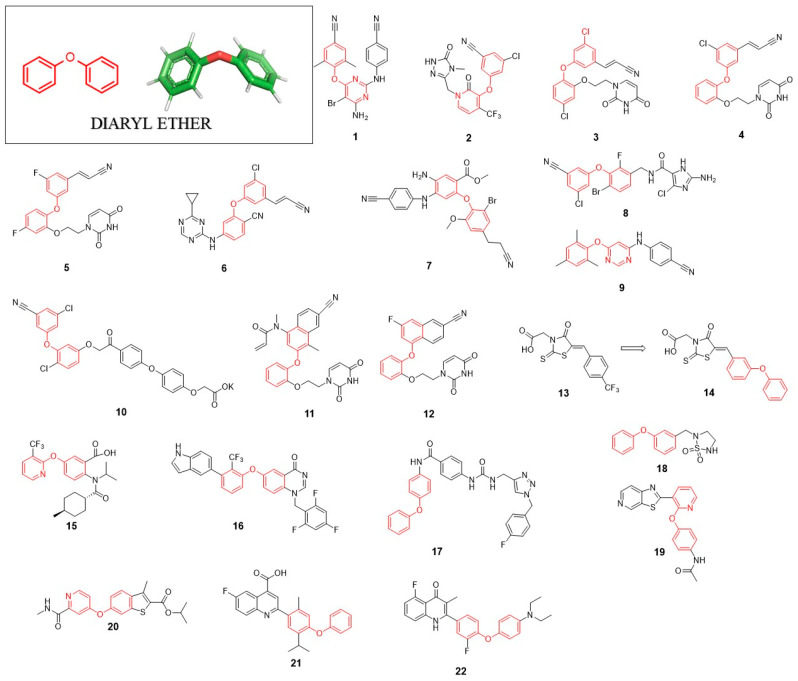
Antivirals with diaryl ether (DE) structure.

Inhibitors targeting HIV-1 reverse transcriptase (RT) are important components of highly active antiretroviral therapy (HAART). The first FDA-approved non-nucleoside inhibitor of HIV-1 reverse transcriptase (NNRTI) was Nevirapine [15]. The potency of this inhibitor’s action is limited in case of single-point mutation (e.g., K103N, Y181C, G190A) variants of HIV-1 reverse transcriptase, which is the reason why extensive research was done to find active, mutation-resistant inhibitors [16]. From six of the FDA-approved inhibitors, Etravirine (TMC125; **1**) and Doravirine (MK-1439; **2**) contain DE with a modified benzene ring within their structure (Figure 2).

Currently, numerous studies are focused on the design and synthesis of HIV reverse transcriptase inhibitors containing a DE motif. Such a biarylether class of inhibitors binds the phenol ring of enzyme Y188 residue to the enzyme through π-stacking of the terminal aryl ring of the inhibitor [17]. In 2011, Bollini et al. presented catechol diether containing uracil and cyanovinylphenyl groups in its structure. Among inhibitors of this series, compound **3** showed extremely high potency of action with EC_50_ and CC_50_ values of 55 pM and 10µM, respectively. The subsequent molecular modelling approach revealed the presence of possible interactions between the phenyl ring of DE and tyrosine side chain 188 [18], which was further confirmed by solving the X-ray crystal structure of the enzyme-inhibitor complex [19]. The continuation of these studies led to the discovery of two inhibitors **4** and **5** which displayed improved potency of action against mutant variants of RT, where compound **4** showed the EC_50_ values of 46 nM and 16nM against Y181C and K103N/Y181C, respectively [20]. Similarly, inhibitor **5** showed EC_50_ = 16 nM and 85 nM toward the same RT mutants. Compound **4** was also more soluble than all examined inhibitors of this class such as Rilpivirine, Nevirapine or Efavirenz, which improved the bioavailability of obtained molecule.

In the same year, Jorgensen et al. developed a series of non-nucleoside reverse transcriptase inhibitors with the structure based on the DE scaffold. From the library of synthesized compounds, inhibitor **6** was most active, showing EC_50_ values of 2.5 nM and 4.9 nM against wild type and emerging mutant Y181C of HIV reverse transcriptase, respectively [21].

In a series of articles published by the Lan Xie research group, diarylaniline was used as a core structure for the development of new HIV-1 non-nucleoside reverse transcriptase inhibitors in which the DE moiety interact with so-called the “western wing” binding pocket of reverse transcriptase [22,23,24,25]. The extensive optimization studies led to the generation of highly potent molecules active against wild type (WT) and E138K mutant reverse transcriptase. Compound **7** displayed low nanomolar EC_50_ values of 5.19 nM and 9.98 nM against WT and E138K mutant, respectively [25].

In 2012, the Andrew Peat group developed extremely potent inhibitors of HIV-1 reverse transcriptase [26]. Compound **8** showed EC_50_ < 1 nM against enzyme wild type as well as against K103N and Y181C mutants. Only a slight activity loss was noticed against Y188L mutant (EC_50_ = 0.5 nM) as compared to the activity against WT. The structure of **8** in addition to the DE motif contains the substituted imidazole ring responsible for extensive H-bond network formation with reverse transcriptase which are of high importance in blocking the activity of Y188L mutant.

In 2012, Ribone et al. presented a series of novel 4,6-diarylpyrimidines and diarylbenzenes as inhibitors of HIV-1 [27]. Among all synthesized derivatives, **9** was the most active against the WT HIV-1 (EC_50_ = 0.049 μM). However, no activity was noticed against the double HIV-1 mutant (K103N + Y181C). Subsequent molecular modelling evaluation revealed that methyl substitutions in the ortho positions of the DE ring introduce steric hindrance responsible for beneficial spatial arrangement, which maximizes the π–π stacking interaction with Y181, Y188 and W229 of HIV reverse transcriptase.

Five years later, Frączek et al. presented a series of novel diaryl ethers with phenacyl moiety as inhibitors of HIV reverse transcriptase [28]. The designed compounds contain a polar group which improves inhibitors solubility. The most promising compound from these studies, **10,** showed the IC_50_ value of 0.36 µM toward HIV reverse transcriptase. Although the authors claimed that **10** showed antiviral activity, unfortunately, no EC_50_ value was provided.

In order to overcome the loss of inhibitors activity toward the Y181C mutation of reverse transcriptase, Chan et al. presented new compounds containing acryl amide warhead able to form a covalent bond with C181 [29]. The most potent inhibitor **11** showed an irreversible mode of action with the k_2_/K_i_ of 195,000 M^−1^s^−1^. Further studies of antiviral activity using HIV-1-infected cells showed that 5 displayed the EC_50_ values of 0.56 µM and 0.5 µM against WT and K103N/Y181C, respectively. Crystallographic data indicate that **11** containing the methylacrylamide group forms a covalent bond with the thiol group of HIV reverse transcriptase C181. Although the EC_50_ value of **11** is not impressive when compared to the already known reverse transcriptase inhibitors, the concept of irreversible inhibition of HIV reverse transcriptase seems to be an attractive alternative for a further development of novel antiviral drugs. The application of the inhibitors of the irreversible type is especially beneficial when applied against drug-resistant mutants.

Recently, Kudalkar et al. have identified an effective HIV-1 NNRTI: **12** was very potent with an IC_50_ value of =4.8 nM and exhibited very potent antiviral activity in a cell-based assay displaying an EC_50_ value of 1.1 nM with a simultaneous lack of cytotoxicity (CC_50_ > 100 µM) [14]. The authors also prepared poly(lactide-coglycolide)-based particles as long-acting nanoformulation of **12**. One of the aryl groups from DE moiety present in this compound was replaced by the naphthyl ring. As crystallographic data show, this naphtyl ring interacts with the enzyme via van der Waals interactions with P95, L100, V108, Y188, W229, F227 and L234 residues. The naphthyl ring formed a π–π stacking interaction with Y188 and W229 residues. The central catechol ring is responsible for the interaction with Y181 via π–π interaction.

### 2.2. Anti-HCV Agents

DE showed itself to be a favourable group in the development of inhibitors targeting RNA-dependent RNA polymerase (RdRp, NS5B). This enzyme plays a crucial role in HCV replication and is an important molecular target used for anti-HCV drugs design. Talele et al. showed that the incorporation of DE moiety into the thioxothiazolidin-type inhibitor (**13**) improved its activity (7-fold more potent inhibitor-compound **14**). It is probably because of the strong π-cation and hydrophobic interactions between DE and NS5B protein [30]. Inhibitor **14** possesses another privileged structure, rhodanine, which is described later.

In 2013, Stammers et al. discovered potent anthranilic acid-based NS5B polymerase inhibitors. Compound **15** with 3-trifluoromethylpyridin-2-yl group introduced into the DE moiety showed low IC_50_ and EC_50_ values [31]. Later, the same group applying the SAR approach discovered compound **16** as a highly potent inhibitor HCV NS5B polymerase with antiviral activity against HCV genotypes 1a and 1b [32]. Compounds **15** and **16** bind into the allosteric site of HCV NS5B polymerase (thumb pocket 2) and the modified DE group is essential for this interaction.

### 2.3. Anti-Flaviviruses Agents

One of the promising anti-FLV agents are inhibitors of NS2B-NS3 protease, which is a key enzyme in the virus replication cycle, responsible for viral polyprotein processing. Benz[d]isothiazol-3(2H)-one-triazoles are a class of inhibitors reported in 2012 by Groutas’s research group. They synthesised para- and meta-substituted aminobenzamide derivatives. Planar aminobenzamide allows fitting the inhibitor into the shallow active site of DENV and WNV proteses; additionally, aminobenzamide structure orienting substituents to interact with multiple active site residues. The studies led to the discovery of inhibitor **17** containing DE moiety in its structure, which was found to be active against both DENV (K_i_ = 8.77 µM) and WNV NS2B-NS3 (K_i_ = 5.55 µM) proteases [33]. Molecular docking studies revealed hydrophobic interactions between the DE group and Val72. Grouta’s group also discovered a potent inhibitor of noroviruses-compound **18**. This heterocyclic compound with DE part showed EC_50_ = 4 µM and is a good starting point for the development of new antinorovirus agents [34].

### 2.4. Anti-Polyomaviruses Agents

Polyomaviruses compose a family of small double-stranded DNA viruses. Although an infection with these viruses does not cause serious diseases, nevertheless, in immunocompromised patients (e.g., patients after kidney transplantation), it can trigger a progressive multifocal leukoencephalopathy or polyomavirus-associated nephropathy. The DE scaffold was found to be beneficial for the development of John Cunningham virus (JCV) and BK virus (BKV) helicase inhibitors. SAR studies led to the discovery of the potent inhibitor **19** of helicase from JCV and BKV. Crystallographic data indicates that the phenoxy group of DE is responsible for the cation−π interaction with Arg555 and Lys419 of helicase. An incorporation of a nitrogen atom into the aromatic ring ensures H-bonds interaction with helicase Lys419. This inhibitor showed antiviral activity in vero cells with an EC_50_ value of 40 µM against JCV with no significant cytotoxicity [35].

### 2.5. Anti-Rhinoviruses Agents

Compound **20** was found to be a potent inhibitor of rhinovirus replication. This small molecule is responsible for the interaction with viral coat protein 1 (VP1) and impedes viral infection at an early stage. Compound **20** inhibited the replication of hRV-B14, A21 and A71 strains with EC_50_ values of 0.083 μM, 0.078 μM and 0.015 μM, respectively. These values are in a range of Pleconaril activity—the antiviral drug used in the common cold symptoms [36]. DE moiety is part of the N-methyl-picolinamide-based inhibitors in which it is crucial for the antiviral effect. As molecular docking studies have shown, N-methyl-picolinamide moiety creates hydrophobic interactions with L106, Y197 and M221 in VP1. Compound **20** exhibit strong inhibition toward poliovirus 3 replication with the EC_50_ of 0.063 µM [37].

### 2.6. Host-Targeting Antivirals

In order to enter the host cell and efficiently replicate inside, viruses use a wide range of host factors that are crucial for virus spread. Thus, more and more attention is directed to the development of inhibitors targeting host enzymes participating in the replication. One of the promising host factors for antiviral intervention is dihydroorotate dehydrogenase (DHODH), an enzyme essential for the synthesis of pyrimidine, crucial for the biosynthesis of DNA, RNA and phospholipids building blocks [38]. Compound **21** is a potent DHODH inhibitor with a low nanomolar IC_50_ value (IC_50_ = 1 nM). This compound showed a broad-spectrum antiviral activity. The cell-based assays showed the IC_50_ value of 2 nM against vesicular stomatitis virus (VSV) and the IC_50_ of 41 nM against influenza A/WSN/33 virus. This inhibitor was developed during the SAR study. The DE group present in the structure of **21** is important for its activity since it creates π–π stacking with the phenylalanine side chain (Phe62) of DHODH. Analogues without the DE group displayed a significant loss of activity against DHODH (260-fold increase of the IC_50_ value) [39].

Another interesting SAR study undertaken by Yang et al. led to **22** as a potent broad spectrum antiviral agent. This quinolone-based DE scaffold-containing compound was found to be a potent DHODH inhibitor with EC_50_ = 0.015 µM. As a strong DHODH inhibitor, the compound **22** showed low IC_50_ values against a wide range of viruses: 4 nM against EV71, 5 nM against HCV, 7 nM against DENV IC_50_ = 13 nM against HIV. The binding mode of **22** to the DHODH is similar to the inhibitor **21**, where the DE group interacts with the aromatic ring of phenylalanine via π–π stacking interaction [40].

## 3. Indole

The indole ring is one of the most important privileged scaffolds employed in drug discovery. A tremendous number of compounds containing this heterocyclic aromatic ring structure are already approved drugs or are currently under clinical trials. In 2015, Zhang et al. published a comprehensive review describing antivirals with indole core [41] while in 2019 and 2020, Kumari et al. and Durababu presented reviews about medicinal chemistry of indole derivatives describing indole containing antivirals [42,43]. Considering the above, we refer to studies published after 2019 in regard to the application of indole ring in antiviral drug discovery. We also briefly present the already approved antiviral drugs containing indole moiety.

**Figure 3:**
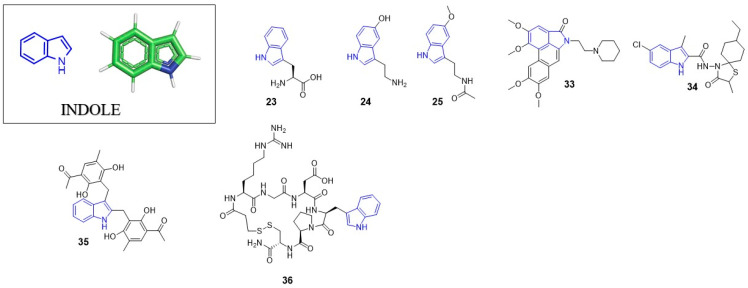
Important compounds (**23–25**) and antivirals (**33–35**) with indole ring.

Indole ring is a pervasive/ubiquitous motif present in many natural products like the side chain of tryptophan (**23**), one of the naturally occurring proteinogenic amino acids, or neurotransmitters including serotonin (**24**), as well as in hormones such melatonin (**25**) (Figure 3). The structure of indole is present in many drugs, including antiviral (Table 1), antibacterial, antimalarial or anti-tumor agents. Such a diversity may by partially explained by relatively easy modification of the indole ring.

### 3.1. Anti-HIV Agents

In 2020, Young Hyun Shin et al. presented the anti-HIV activity of aristolactam derivatives based on the dibenzo[cd,f]indol-4(5H)-one structure [50]. The most active compound from the studies (**33**) showed the IC_50_ value of 1.03 µM (CC_50_ = 16.91 µM). Interestingly, compound **33** is not an HIV-1 reverse transcriptase inhibitor but impedes Tat-mediated viral transcription. This pathway have been already targeted by different class of inhibitors [51,52,53]. The findings of Young Hyun Shin et al. suggest that aristolactams derivatives can be considered as an interesting starting point for the development of a novel class of anti-HIV-1 agents which inhibit the HIV-1 Tat-mediated transcription.

### 3.2. Anti-Influenza Agents

Recently, the Naesens group developed inhibitors of the membrane fusion of the influenza virus [54]. The inhibitor with this mode of action—umifenovir (**26**)—is currently used in influenza treatment. Presented compounds contain an indole ring within the spirothiazolidinone structure. The most potent inhibitor from these studies (**34**) showed an EC_50_ value of 1 nM against influenza A/H3N2 virus with a cytotoxicity CC_50_ = 1.5 µM. Molecular docking studies revealed the possible conformations of the inhibitor. The positively charged indole nitrogen of **34** interacts with solvent via electrostatic interactions and the charged residues present in the enzyme binding pocket, such as E57_2_ of monomer 1.

Recently, the concept of dual inhibitors, also known as designed multiple ligands (DMLs), is attracting great attention in the scientific community. The DMLs concept was introduced by Richard Morphy and Zoran Rancovic [55,56]. Among other things, such an unconventional approach was used during anti-HIV agent development [57,58]. As representative examples could serve dual inhibitors of Tat-mediated transcription and reverse transcriptase [59]. Bifunctional inhibitors of reverse transcriptase and integrase inhibitors are also known [60]. In 2020, Guangwei Wu et al. proposed anti-influenza A virus (IAV) agents displaying a dual inhibitory activity: blocking hemagglutinin-mediated adsorption of the virus particle from one side, and blocking the process of membrane fusion from the other side [61]. From a series of fifteen compounds, inhibitor **35** was found as a potent and broad-spectrum anti-IAV agent. In a single-cycle, replication assay, **35** showed the IC_50_ values of 6.3 µM, 5.5 µM and 9.5 µM against H1N1, H3N2, H1N1 virus subtypes, respectively. In the same assay, umifenovir (**26**), known as the influenza virus fusion inhibitor, displayed the IC_50_ value of 13.3 µM. The structure of **35** is built on the central indole ring scaffold substituted at C-2 and C-3 positions. This is the first IAV inhibitor targeting both HA1 and HA2 subunits of virus hemagglutinin; thus, it is able to block both the IAV adsorption and membrane fusion. Molecular dynamic simulations showed that the indole ring is oriented to the center of the binding site forming T–π interactions with tryptophan side chain (W14) of monomer 1. This compound could be considered as the proof of the concept that drugs which inhibit multiple hemagglutinin functions are effective for therapy of influenza infections.

### 3.3. Anti-Alphavirus Agents

In 2020, Fatma et al. reported Eptifibatide Acetate (**36**) as a potent chikungunya virus capsid protease (CP) inhibitor [62]. This compound also suppressed the viral replication in a cell-based assay showing an EC_50_ value of 4.01 µM. Considering the CP substrate specificity at P1 position toward tryptophan residue, the indole ring present in the eptifibatide structure most likely fits the S1 pocket of the protease.

## 4. 2-(1,3-Oxazinan-3-yl)ethan-1-amine and 2-(Piperidin-1-yl)ethan-1-amine

### 4.1. Anti-HIV Agents

**Figure 4:**
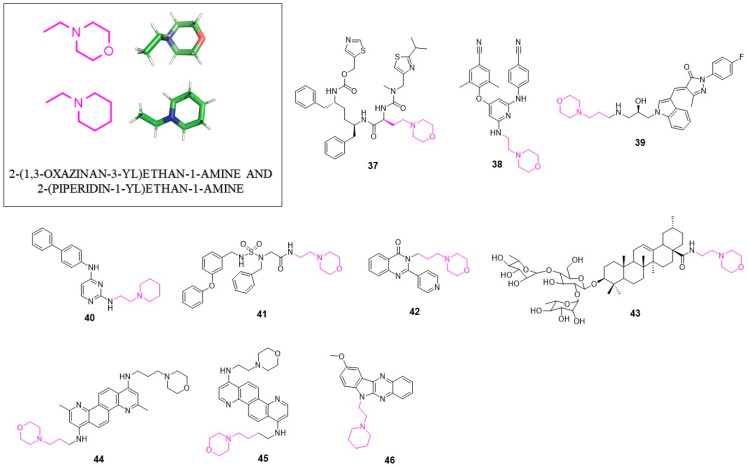
Antivirals with 2-(1,3-oxazinan-3-yl)ethan-1-amine and 2-(piperidin-1-yl)ethan-1-amine.

A representative example of an antiviral agent containing the 2-(1,3-oxazinan-3-yl)ethane structural motif is Cobicistat (GS-9350; **37**, Figure 4) developed by Gilead Sciences. GS-9350 is the inhibitor of human cytochrome P450 3A (CYP3A) used in HIV-1 infection treatment [63].

Compounds with 2-(1,3-oxazinan-3-yl)ethan-1-amine moiety were found to be useful NNRTIs against mutant variants of HIV-1 reverse transcriptase [64], in particular, against K103N mutation. Compound **38** showed EC_50_ = 49 nM, which is a significantly higher activity when compared with other known NNRTIs (EC_50_ = 6.78 μM for NVP; EC_50_ = 2.48 μM for DLV and EC_50_ = 0.12 μM for EFV). 2-(morpholinoethyl)amino group forms a hydrogen bond with Lys101, which probably plays an essential role in improving activity against the K103N mutant strain. These results highlight great potential of this lead compound for further optimization of new anti-HIV-1 agents.

### 4.2. Anti-HCV Agents

In 2016, Han et al. developed several novel HCV inhibitors which block the step of virus entry [65]. The SAR studies identified compound **39** which displayed good anti-HCV activity (EC_50_ = 0.72 μM) and high selectivity (SI > 69.44) towards the cytotoxicity effect. Interestingly, in addition to 2-(1,3-oxazinan-3-yl)ethane, unit inhibitor **39** contains an indole ring, previously described as a privileged structure. The potency of compound **39** encourages the development of new anti-HCV agents based on its structure.

### 4.3. Anti-Flavivirus Agents

In 2019, a set of novel compounds targeting DENV E protein was presented [66]. The *de novo* design followed by molecular dynamics optimization led to **40**, which was the most potent compound of the series active against all four serotypes of DENV (DENV1–DENV4) with the EC_50_ value of 0.87 μM; 0.85 μM; 0.56 μM; 2.5 μM against DENV1, DENV2, DENV3, DENV4, respectively, and CC_50_ = 18.1 μM and a satisfactory in vitro pharmacokinetics profile with solubility in simulated gastric fluid 506 µg/mL and t_1/2_ > 120 min.

### 4.4. Anti-Norovirus Agents

In 2012, the synthesis of acid-derived acyclic sulfamide-based inhibitors of norovirus was reported [67]. From a diversified library of compounds, **41** was the most potent with the EC_50_ value of 3.8 µM and the TD_50_ value of 45 µM in a cell-based replicon assay. Interestingly, the mechanism of action of this type of inhibitors remains unknown.

### 4.5. Anti-Influenza Agents

Liu et al. developed new 2-pyridinyl-3-substituted-4(3H)-quinazolinones as anti-influenza A virus agents [68]. Compound **42** was one of the most potent with the IC_50_ value of 54 µM against H1N1. Further studies of its mechanism of action revealed that this class of inhibitors impedes virus neuraminidase activity as well as the cellular NF-*κ*B signaling pathway.

In 2019, Hui Li et al. described new entry inhibitors of influenza virus [69]. An incorporation of 2-(piperidin-1-yl)ethan-1-amine and 2-(1,3-oxazinan-3-yl)ethan-1-amine structures into 3-O-β-chacotriosyl ursolic acid via an amide bond resulted in the generation of novel IAV inhibitors. Compound **43** showed the IC_50_ values of 4.13 µM, 18.18 µM and 20.12 µM against H5N1, H1N1 and H3N2, respectively. Most probably, **43** is able to bind to the viral hemagglutinin (HA). The molecular docking studies showed that **43** fits into the conserved pocket in the trimeric form of HA. Considering its activity, compound **43** is an excellent lead structure for further development of more potent anti-IAV agents.

### 4.6. Anti-Ebola Agents

Compound **44** is a symmetrical molecule containing two 2-(1,3-oxazinan-3-yl)ethan-1-amine groups in its structure [70]. It was discovered during the work focused on new *Plasmodium falciparum* inhibitors. It showed in vitro the IC_50_ value of 2.5–5 µM in a EBOV assay. Possibly this compound targets some host cellular pathways important for virus replication. Furthers studies are needed to confirm this hypothesis. Further studies revealed that analogous compound to **44** inhibitor **45** administered at a low dose (10 mg/kg/day) showed in mouse models 100% protection against Ebola virus [71]. In the HeLa cells, assay **45** displayed the EC_50_ value of 0.26 µM.

### 4.7. Anti-Vaccinia Virus Agents

Vaccinia virus (VACV) is a double-stranded DNA virus of the *Poxviridae* family. Its similarity to the variola virus (smallpox) makes it a good model to use for evaluation of novel agents targeting serious potential biothreat. The virtual screening of diversified combinatorial library led to the identification of potential nucleic acid intercalators with anti-vaccinia activity [72]. The most promising compound (**46**) inhibits virus reproduction with the IC_50_ value of 5 µM and SI = 34 (SI = CC_50_/IC_50_). These findings could serve as a starting point for the development of broad-spectrum antivirals of mechanism based on nucleic acid intercalation.

## 5. Artemisinin (ART)

**Figure 5:**
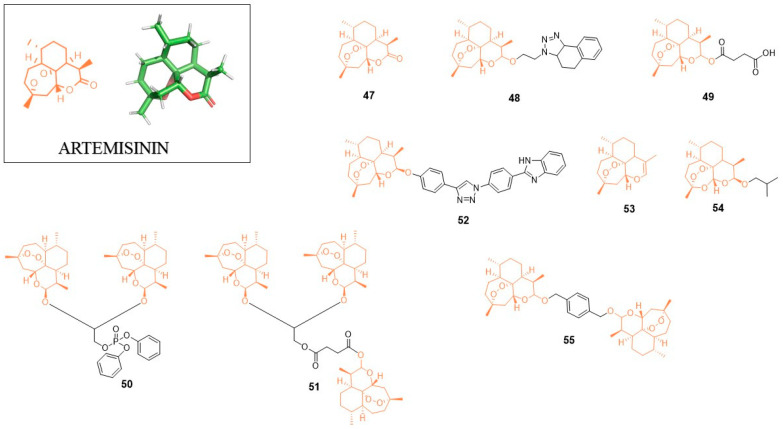
Artemisinin derivatives with antiviral activity.

Artemisinin (ART; **47**, Figure 5) is a natural product derived from the Chinese herb *Artemisia annua*. Originally, this compound was identified as a strong anti-malaria agent winning for Youyou Tu the 2015 Nobel Prize in Physiology or Medicine for key contributions to the discovery of artemisinin [73]. Apart from its anti-malaria activity, artemisinin shows anticancer activity. Interestingly, the bioactivity of artemisinin and its derivatives encompasses also inhibition of viruses including the *Herpesviridae* family (human cytomegalovirus, herpes simplex virus type 1 or Epstein-Barr virus), hepatitis B and hepatitis C [74,75].

### 5.1. Anti-HIV Agents

In 2017, Jana et al. synthesized a library of fused 1,5-disubstituted 1,2,3-triazole artemisinin derivatives [76]. The most active inhibitor **48** showed an IC_50_ value of 2.78 µM against HIV-1 III_B_. Although this activity is significantly higher when compared to Etravirine, which showed the IC_50_ value of 3.4 nM in the same assay, the authors postulated that the developed compound acts as a first generation NNRTI since it lacks the activity against the double reverse transcriptase mutant (K103N; Y181C) of HIV-1 (strain RES056) with IC_50_ > 31.5 µM.

### 5.2. Anti-Flavivirus Agents

Recently, the Jing Ye research group discovered that artemisinin (**47**) could be useful as a therapeutic agent able to inhibit the replication of flaviviruses such as Japanese encephalitis (JEV), Dengue virus (DENV) and Zika virus (ZIKV) [77]. The authors also investigated the mode of artemisinin action showing that upon viral infection, ART enhances the host type I interferon production. Animal-based studies showed that JEV-infected mice treated with ART showed the reduction of viremia, neuroinflammation and mortality. Taken together, ART could be advantageous as a broad-spectrum inhibitor of flaviviruses.

### 5.3. Anti-Herpesvirus Agents

Artesunate (ARS; **49**) is a synthetic derivative of the natural artemisinin used in malaria treatment, which displays activity against human cytomegaloviruses [78,79]. Sunwen Chou et al. described new discoveries in regard to anti-CMV activity of ART [80]. They showed that ART is able to block CMV standard therapy-resistant virus mutants with a 2-fold range compared to wild-type virus (e.g., IC_50_ = 3.26 µM for WT vs. IC_50_ = 6.25 µM for L595S mutant in HEL cells). They also found that ART treatment of HCMV-infected cells after virus adsorption enhance antiviral activity. ART showed a synergistic effect in combination with inhibitor of viral protein kinase maribavir (MBV).

In 2011, Ran He et al. found that artemisinin-derived dimers are more potent inhibitors of human cytomegalovirus (CMV) replication than artemisinin-derived monomers [81]. The most active compound from the studies **50** containing diphenyl phosphate (DPP) function showed EC_50_ = 0.04 µM with CC_50_ = 55.8 µM, while in the same assay, the reference compound ganciclovir displayed EC_50_ = 2.7 µM with CC_50_ = 247 µM. Compound **50** showed the strong inhibitory effect on the growth of cancer cell lines (HeLa, 1205Lu, HCT116, HFF). Later, the same research group tried to optimize the **50** structure. The replacement of phenyl rings in diphenyl phosphate moiety resulted in activity loss. Only the molecule with the dicyclohexylphosphate unit showed activity comparable to that of the lead compound (EC_50_ = 0.044 µM) [82]. Further studies on **50** showed that this dimer is an irreversible CMV inhibitor. Interestingly, the analog devoid of the peroxide unit within the artemisinins structure showed lowest anti-CMV activity [83].

In 2015, Reiter et al. developed the artemisinin-derived trimer (**51**) without DPP function which displayed a IC_50_ value of 0.04 µM, very similar to the DPP artemisinin dimer [84]. Compound **51** showed high potency against CCRF-CEM and CEM/ADR5000 leukemia cell lines with activity greater than the reference compound, i.e., doxorubicin.

Recently, the monomeric anti-CMV derivative of artemisinin containing a triazole unit in its structure was reported [85]. Compound **52** showed EC_50_ = 0.26 µM and low cytotoxicity (CC_50_ > 60 µM) while its autofluorescent properties helped to localize it inside the cell. BF95 accumulates in the mitochondria and reduces mitochondrial membrane potential. The analogue compound without the endoperoxide bridge did not show any antiviral activity and had no impact on mitochondrial membrane potential. This observation suggests the importance of mitochondria in the antiviral mechanism of artemisinin derivatives action.

### 5.4. Anti-HBV Agents

In 2005, the anti-HBV properties of ART and ARS were demonstrated on the in vitro model of HepG2 2.2.15 [46], which led to the discovery of new artemisinin derivatives with potential antiviral properties [86]. From eight new compounds, inhibitors **53** and **54** were able to reduce the release of HBV-DNA to the medium at non-toxic concentrations in HepG2 cells permanently infected with hepatitis B virus.

### 5.5. Anti-HCV Agents

ART was found to inhibit the HCV replication in vitro at concentrations that have no effect on host cell growth [87]. Later, ART analogs with enhanced activity were developed. Studies presented by Obeid et al. showed that inhibitor **55** is a potent anti-HCV agent in a/the Huh 5-2 HCV replicon assay with EC_50_ = 3.2 µM and CC_50_ > 133 µM [88]. Studies with carbon-centered radicals-trapping compound revealed that radicals are not the main effectors of the anti-HCV activity of the artemisinin derivatives. A possible mechanism of action of this compound includes the induction of reactive oxygen species (ROS).

## 6. 1,3,4-Oxadizole

1,3,4-oxadiazole is a heterocyclic five-membered ring which is an important feature in new compounds, with numerous biological activities, such as anti-cancer, anti-inflammatory, anti-bacterial, anti-protozoal and anti-viral. Compounds containing the 1,3,4-oxadiazole scaffold are known anti-HIV, anti-HCV, anti-HBV, anti-HAV and anti-HSV-1 agents. In 2011 and 2017, two excellent reviews about the chemistry and biology of 1,3,4-oxadiazoles were published [89,90]. Herein, we supplement the information present in the mentioned articles.

### 6.1. Anti-HIV Agents

**Figure 6:**
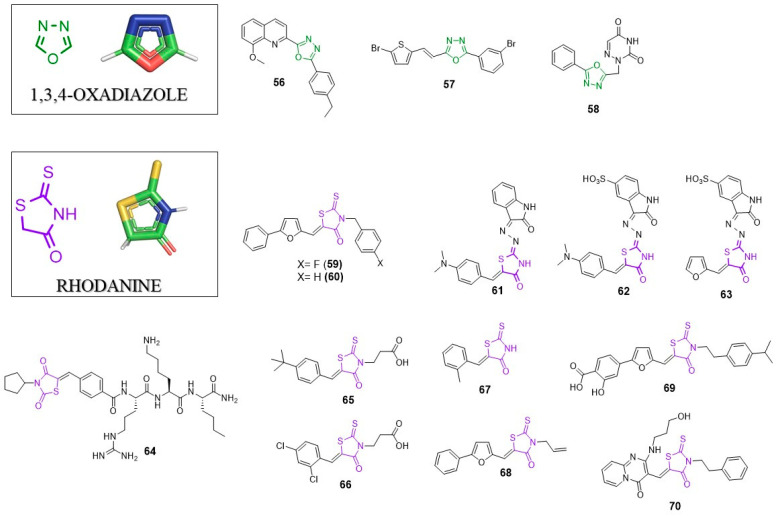
1,3,4-Oxadiazole and rhodanine containing antivirals.

In 2018, Shah et al. showed results about new quinolines as anti-HIV agents. As the part of the work, they synthesized the 1,3,4-oxadiazole-quinoline hybrid. One of the compounds (**56**, Figure 6) showed IC_50_ = 45 µM and 135 µM against HIV-1_VB59_ and HIV-1_UG070_, respectively, with low cytotoxicity (CC_50_ = 413.38 µM) [91]. Better activity was observed for derivatives with *α*,*β*-unsaturated amide moiety instead of 1,3,4-oxadiazole. The most active compound containing this structure displayed IC_50_ = 3.35 µM (against HIV-1_VB59_) and 2.57 µM (against HIV-1_UG070_).

### 6.2. Anti-Flavivirus Agents

1,3,4-oxadiazole derivatives were found to be potent anti-flavivirus agents. High-throughput screening and further SAR studies helped to develop potent inhibitors of viral NS5 polymerase [92]. The most active inhibitor with 1,3,4-oxadiazole moiety (**57**) showed IC_50_ = 4.0 µM in in a DENV-2 RdRp assay and EC_50_ = 4.9–9.1 µM against four DENV serotypes (DENV1–DENV4) with not cytotoxic effect in a Vero E6 cells assay (CC_50_ > 100 µM).

### 6.3. Anti-Herpesvirus Agents

Recently, El Mansouri et al. designed and synthesized a series of 1,3,4-oxadiazole-pyrimidines and purines hybrids [93]. One of the compounds (**58**) showed a great potency of action against thymidine kinase-negative strain (TK-VZV) with EC_50_ = 50 µM, which is two times better than the activity of Acyclovir (EC_50_ = 103 µM) and comparable with Brivudine (35 µM).

## 7. Rhodanine (RHO)

Rhodnanine (2-thioxothiazolidin-4-one) is a 5-membered heterocyclic structure present in several biologically active compounds including antibacterial, anticancer and antiviral agents [94]. Possible modifications of RHO structure are practically unlimited, which yields a great number of its derivatives. Rhodanine is most often substituent in 5 position; however, the introduction of a/the substituent in different positions is possible.

### 7.1. Anti-HIV Agents

In 2011, Botta’s research group performed an optimization of the previously found inhibitor of HIV integrase [95,96], which led to the discovery of a series of potent integrase inhibitors able to inhibit HIV-1 replication. Compounds **59** and **60** were the most potent derivatives displaying IC_50_ = 6.9 µM; EC_50_ = 1.7 µM for **59** and IC_50_ = 3.7 µM and EC_50_ = 8.2 µM for **60**. Molecular docking studies showed the existence of hydrophobic interactions between the rhodanine substituent of **59** and Y143 of HIV integrase.

The rhodanine scaffold is also found in nucleocapsid protein 7 (Ncp7) inhibitors. Ncp7 is a small nucleocapsid protein with the basic character. Since it is crucial for the viral replication at several stages and is found in all viral strains, Ncp7 is considered an attractive therapeutic target [97,98]. Researchers from Boehringer Ingelheim identified three potent rhodanine based Ncp7 inhibitors with IC50 values: 0.59 µM; 0.11 µM; 0.95 µM for **61**, **62** and **63**, respectively [99]. The NMR based studies revealed the binding mode of inhibitor 3. Ncp7 create two inhibitor binding sites (A and B). One molecule of the inhibitor binds to the A binding sites while the second inhibitor molecule interacts mainly with the B binding site and becomes a linker of these two binding sites. The rhodamine moiety of one inhibitor molecule interacts with the furan ring of the second inhibitor.

### 7.2. Anti-HCV Agents

Rhodanine was found to be a favourable scaffold in the development of NS5B HCV polymerase inhibitors. In 2010, Talele et al. performed a virtual screening of 260,000 compounds and found the rhodanine scaffold-based molecule as good starting point for further SAR exploration and modification [30]. From all synthesized compounds, derivative **14** was the most potent inhibitor of NS5B with IC_50_ = 4.26 µM. Molecular docking suggests the importance of the rhodamine scaffold in the interaction with viral enzyme; the C_4_ carbonyl oxygen atom of the rhodanine ring creates a hydrogen bond with the guanidine group of Arg503 of enzyme. The heterocyclic ring is stabilized by the side chains of Pro495 and W500. It is worth noting that compound **14** contains a DE unit which is another privileged scaffold discussed earlier.

### 7.3. Anti-Flavivirus Agents

As the continuation of the studies on the inhibitors of NS2B-NS3 protease, Klein’s research group developed amide type thiazolidinedione-capped and rhodanine-capped inhibitors [100]. Generally, inhibitors with thiazolidinedione moiety showed better activity in an/the enzymatic assay; however, rhodanine-capped peptide hybrids had better antiviral activity in cell culture than the thiazolidinediones. The most potent rhodanine containing inhibitor (**64**) showed IC_50_ = 6.1 µM and K_i_ = 9.3 µM, while in antiviral assay, **64** had EC_50_ = 16.7 µM.

In 2012, Stahla-Beek reported the first inhibitors that target the guanylyltransferase activity of the flavivirus NS5 RNA capping enzyme with antiviral properties [101]. HTS identified a series of rhodamine containing low molecular weight inhibitors of the dengue virus NS5 RNA capping enzyme. Further SAR analysis led to **65** (BG323), which was able to efficiently inhibit the West Nile virus and yellow fever virus in cell-based assays. **65** showed Ki = 7.5 µM and 9.5 µM against dengue virus and yellow fever virus capping enzymes, respectively. EC_50_ for this compound in a dengue virus replicon antiviral activity assay reached 30.8 µM with relative low cytotoxicity (CC_50_ = 184 µM). Interestingly, another inhibitor, **66** (BG330, Ki = 9.8 µM and 9 µM against dengue virus and yellow fever virus, respectively), in a cell-based assay showed EC_50_ = 2.6 µM, which is almost 12 times better than BG-323. Unfortunately, the cytotoxicity of BG330 was significantly higher than BG323 (CC_50_ = 12 µM). Molecular docking showed that central rhodamine adjusts substituents into enzyme binding pockets and possible hydrogen bond formation between Lys13 and oxygen atom of rhodanine.

Recently, Quek et al. used a fragment screening approach in order to search for novel anti-ZIKV protease agents [102]. This approach is one of the commonly used techniques in drug development. Screening of the compound library allowed the identification of low molecular ligands whose bonding modes are determined by X-ray crystallography or NMR spectroscopy. Further structures optimization allow highly active molecules [103,104]. Quek at al. found that the compound that was efficiently bound to the S1 pocket of the enzyme was rhodanine. Crystallographic data showed an interaction between rhodaniene and the enzyme. The hydrogen bond between oxygen of RHO and S135 as well as between the amide group and the hydroxyl group from Y130 were identified. These data suggest that the rhodanine scaffold can be used as a starting point for further development of more potent inhibitors of ZIKV protease.

### 7.4. Anti-Alphavirus Agents

In 2015, Jadav et al. synthesized and studied the antiviral activity of new rhodanie derivatives [105]. Five compounds from the studies were identified as active inhibitors of chikungunya virus. The most potent derivative **67** showed IC_50_ = 0.42 µM. Molecular docking suggests that compound **67** may act as an nsp2 CHIV protease inhibitor. Most probably, the rhodamine ring fits into the S2 pocket while aryldiene substituent occupies the S3 pocket. Further studies are necessary to verify this assumption.

### 7.5. Broad Spectrum Antivirals

Compound **68** is known as an antiviral molecule active against various enveloped viruses including influenza A, filoviruses, poxviruses, arenaviruses, bunyaviruses, paramyxoviruses, flaviviruses and HIV-1 [106]. Compound **68** contains an allyl moiety in 3 position and 2-phenylofuryl group substituted in 5 position of rhodamine moiety. SAR studies showed that both groups are essential for the compound’s activity. It showed IC_50_ = 1 µM against nipah virus in a neutralization assay. The broad-spectrum activity is the result of its mechanism of action. It inhibits viral entry at a step after virus binding to the cell but before virus cell fusion via intercalation into the viral membrane. Further studies revealed the exact molecular mechanism of **68**′s action. Compound **68** induces viral membrane lipid oxidation by singlet oxygen (^1^O_2_) generation. This event negatively impacts the viral membrane and impedes virus-cell fusion. Since the mechanism of action of **68** is strongly dependent on light, its use in antiviral therapy is of limited application [107,108].

In 2018, Cagno et al. presented studies on the development of compounds similar to LJ001 [109,110]. The most potent compound was derivative **69** with EC_50_ = 6.55 µM against HSV-2 and EC_50_ = 1.6 µM against HSV-2 acyclovir-resistant strain. Derivative **69** was also active against other enveloped viruses such as HSV-1, HCMV, RSV, ZIKV, IAV and VSV remaining inactive against non-enveloped viruses. These data suggest a mechanism of action similar to that of LJ001 and cast considerable doubt on its application of **69** in antiviral therapy.

### 7.6. Host Factors–Targeting Antivirals

One of the most essential host factors required for viral replication is the DEAD-box RNA helicase/ATPase DDX3. Maga et al. developed new DDX3 inhibitors with active in enzyme assay and in infected cells [111]. Compound **70** was the most active against target DDX3 enzyme showing K_i_ = 0.2 µM able to suppress HIV-1 replication (EC_50_ = 6.5 µM). Although the cytotoxicity of **70** is significant (CC_50_ = 65 µM), this compound might be a useful template to design more specific DDX3 inhibitors.

## 8. Pyrrolo[2,3-d]pyrimidine (7-deazopurine) Nucleoside

This group of compounds mimics purines (occurring in DNA and RNA). As such, it could replace purine nucleosides in DNA and RNA. 7-deazapurine structure could be modified in a variety of ways including C6, C7, C8 substitution as well as the generation of fused heterocyclic rings. Such heterocyclic unit can provide efficient π–π or cation–π stacking interactions between active molecule and target protein. In 2017, Perlikova and Hocek published a review article describing antitumor and antiviral properties of 7-deazapurines [112]. Compounds with the 7-deazapurine scaffold are potent inhibitors of RNA-dependent RNA polymerase (NS5B) with anti-HCV activity. 7-deazapurine nucleosides effectively inhibit viruses of the Flaviviridae family (DENV, ZIKV, WNV, YFV), herpesviruses, HBV, HRV-C or EV71.

**Figure 7:**
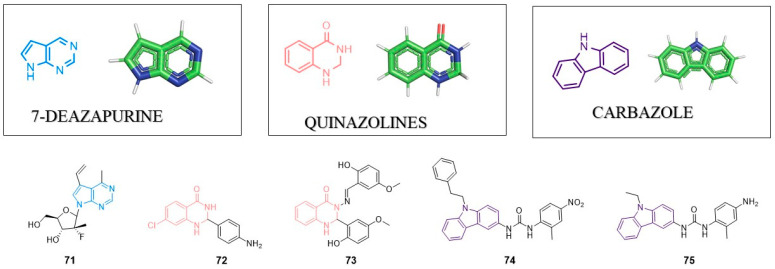
Examples of antivirals wit 7-deazapurine, quinazoline and carbazole moiety.

Recently, Lin et al. designed and synthesized a series of novel 7-deazapurines displaying anti-DENV properties. Inhibitor **71** showed EC_50_ = 2.08 µM and CC_50_ = 150.06 µM [113] (Figure 7). Inhibitor **71** was active against four serotypes of DENV (DENV1–DENV4), which suggests that the target of these compounds is highly conserved among different DENV types. Most likely, compound **71** inhibits RNA polymerase, though further studies are needed to confirm this hypothesis.

## 9. Quinazolines

Derivatives of these heterocyclic scaffolds/this heterocyclic scaffold are known due to a wide range of biological activity including anti-viral properties. Quinazolines could be divided into two groups: quinazolin-2(1H)-one and quinazolin-4(3H)-one. In 2018, Alagarsamy et al. provided a comprehensive review about quinazolines and their pharmaceutical activity [114]. The quinazoline scaffold is present in inhibitors of HIV-1, HIV-2, HSV-1, CMV, JEV, influenza virus and vaccinia virus.

### 9.1. Anti-HIV Agents

In 2014, Modh et al. presented synthesis and biological activity of quinazoline–triazine derivatives [115]. However, these compounds showed high cytotoxicity in MT-4 cells and no specific anti-HIV activity was found for quinazoline–triazine derivatives.

Sancineto et al. performed virtual screening and identify quinazoline as ligand of cyclin-dependent kinase 9 (CDK9), which is critical for HIV-1 Tat-mediated transcription [116]. The most potent inhibitor (**72**) showed IC_50_ value 26.2 µM against CDK9 and inhibits HIV reactivation from latently infected cells with IC_50_ = 4.0 µM. Compound **72** showed no significant cytotoxicity in MT4 and OM-10.1 cells. These data suggest that compound **72** with quinazoline scaffold can serve as the template for further optimization.

### 9.2. Anti-HCV Agents

In 2019, Rothan et al. developed new quinazoline based inhibitors of HCV NS3-4A protease showing antiviral activity in a Huh-7 cells-based assay [117]. NS3-4A serine protease is known as a molecular target in anti-HCV therapy and inhibitors of this enzyme are already approved drugs. Inhibitor **73** was the most potent inhibitor of NS3-4A with IC_50_ = 42 µM. In HCV replicon assay, **73** showed the reduction of Rluc activity at 40 µM (over two times activity loose in Rluc activity). A molecular docking study revealed a possible bonding mode of **73**. Heterocyclic quinazoline moiety crates π–π stacking interaction with enzyme. Although the activity of compound **73** is much weaker than that of the already known inhibitors, it could be considered as a good starting point for further optimization and the development of new class of anti-HCV agents.

## 10. Carbazole

Heterocyclic carbazole with two benzene rings fused with a five-membered nitrogen-containing ring scaffold is present in the structure of several antiviral agents active against HIV, HCV, HCMV, HSV or human papilloma viruses (HPVs). In 2019, Caruso et al. published a review article about carbazole derivatives with antiviral activity [118]. Just to supplement this excellent review, at the end of 2019, Spizzichino et al. presented a series of novel molecules based on a carbazoyl-aryl-urea structure which were potent Zika virus NS5 methyltransferase able to suppress virus replication [119]. SAR studies led to the creation of compound **74** as the most active inhibitor of ZIKV-NS5MTase with IC_50_ = 23 µM in an enzyme-based assay. This compound did not show antiviral activity in cell-based assay. Moreover, the most active compound against ZIKV with EC_50_ of 1.67 μM in cell-based assay (**75**) displayed poor inhibitory activity toward the NS5-MTase in vitro. Further studies are required to unravel these unclear results. A possible explanation is that compound **75** targets another viral enzyme different from NS5 methyltransferase.

## 11. Conclusions

In this review, we presented privileged structures in known anti-viral agents (Table 2). It is absolutely clear that particular motifs of organic compounds are particularly beneficial to the development of antivirals. Privileged structures displayed versatile binding properties and are able to provide potent and selective interaction with a range of different biological targets. Privileged structures provide good drug-like properties. The result of incorporation of the privileged structure into the molecule is the production of high quality leads ready for further drugs development. Since privileged structures provide active and selective ligands for multiple biological targets, their application helps to simultaneously search for clinical candidates in multiple therapeutic fields. It accelerates the research and development of novel candidate drugs. A possible approach with privileged structure application is the modification of exciting bioactive compounds or compound libraries with certain PS. This could lead to the structures with new biological activity. Although a lot of data are available, already more information about the mode of action of a particular compound is necessary to verify its usefulness. For instance, artemisinin derivatives could be active against a wide range of viruses because of their ability to induce the antiviral host factor production instead of interaction with different viral enzymes.

Another problematic issue is the existence of PAINS. One of the discussed privileged structures, rhodamine, is also a known PAINS. Despite the fact that rhodanine containing compounds is the subject of numerous studies, no rhodamine derivative has entered clinical trials [120]. Misidentification of the true hit leads to the waste of time and money to optimize the activity of wrong compounds.

Taken together, we predict that studies on exciting privileged structures as well as the identification of new advantageous scaffolds are only a matter of time. However, researchers should carefully check the real activity of the possible hits to avoid confusion with PAINS.

## Figures and Tables

**Table 1:** Accepted antivirals with indole ring.

Name	Structure	Activity	Molecular Target	Status	References
Umifenovir	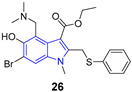	Anti-Influenza	Fusion inhibitor	Approved *	[44]
Delavirdine	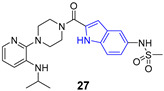	Anti-HIV	Reverse transcriptase	Approved	[45]
Enfuvirtide	AcFTSLIHSLIEESQNQQEKNEQELLELDKWASLWNWF-CONH_2_ (**28**)	Anti-HIV	Fusion inhibitor	Approved	[45]
Elbasvir	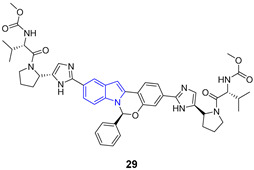	Anti-HCV	NS5A inhibitor	Approved	[46]
Atevirdine	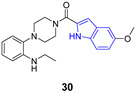	Anti-HIV	Reverse transcriptase	Phase I	[47]
BILB 1941	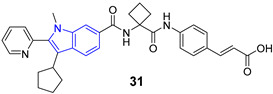	Anti-HCV	NS5B polymerase	Phase I	[48]
Beclabuvir	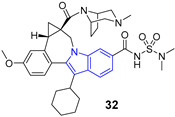	Anti-HCV	NS5B polymerase	Phase II	[49]

* Drug approved only in the Russia and China.

**Table 2:** Privileged structures present in antiviral compounds.

			Diaryl Ether	Indole	2-(1,3-Oxazinan-3-yl)ethan-1-amine	Artemisinin	1,3,4-Oxadiazole	Rhodanine	7-Deazapurine	Quinazolin	Carbazole
**Virus and Molecular Target**	HIV	Reverse transcriptase	[17,18,19,20,21,22,23,24,25,26,27,28,29]	[42,43]	[64]	[76]	[89,90]			[114]	[118]
		Integrase						[95,96]		[104]	
		Fusion		[42,43]							
		Ncp7						[99]			
		Tat		[50]						[116]	
		Unknown		[42]			[91]			[114]	[118]
	HCV	NS3-4A protease								[117]	
		NS5B polymerase	[30,31,32]	[42,43]			[87]	[30]	[112]		[118]
		NS5A protein		[41]							
		Virus entry			[65]						
		Unknown				[70,71]					[118]
	HBV	Unknown				[86]			[112]		
	FLV	NS2B-NS3 protease	[33]					[100,102]			
		RNA polymerase					[92]		[112]		
		E protein			[66]						
		NS5 RNA capping enzyme						[101]			
		NS5 methyltransferase									[119]
		Interferon production enhancement				[77]					
		Unknown							[113]	[114]	
	IAV/IBV	Fusion		[54,61]	[68]						
		Neuraminidase		[61]	[69]						
	Herpesviruses	Viral kinase								[114]	
		Unknown				[78,79,81,82,83,84,85]	[93]		[112]	[114]	[118]
	Polyomaviruses	Helicase	[35]								
	Rhinoviruses	Viral coat protein (VP1)	[36,37]								
		Unknown							[112]		
	Alphaviruses	Capsid protease		[62]							
		NSP2 protease						[105]			
	Adenoviruses	Unknown								[114]	
	Noroviruses	Unknown	[34]		[67]						
	HPV	Unknown									[118]
	Ebola virus	Unknown			[70,71]						
	Vaccinia virus	Nucleic acid intercalation			[72]						
	Host target	dihydroorotate dehydrogenase	[38,39,40]								
		cytochrome P450			[63]						
		DEAD-box RNA helicase/ATPase DDX3						[111]			

## Data Availability

No new data were created or analyzed in this study. Data sharing is not applicable to this article.
